# Co-location of out of hours primary care and emergency department in Belgium: patients’ and physicians’ view

**DOI:** 10.1186/s12913-021-06281-y

**Published:** 2021-03-26

**Authors:** Birgitte Schoenmakers, Jasper Van Criekinge, Timon Boeve, Jonas Wilms, Chris Van Der Mullen, Marc Sabbe

**Affiliations:** Department of Public Health and Primary Care, KU Leuven, Kapucijnenvoer 33 box 7001, 3000 Leuven, Belgium

**Keywords:** Family practice, Health services research, Quality development, Emergency medical services, Triage

## Abstract

**Background:**

In Belgium, General Practitioner Cooperatives (GPC) aim to improve working conditions for unplanned care and to reduce the number of low acuity emergency visits. Although this system is well organized, the number of low acuity visits does not decrease.

**Methods:**

We explored the view of patients and physicians on the co-location of a GPC and an emergency service for unplanned care. The study was carried out in a cross section design in primary and emergency care services and included patients and physicians. Main outcome measure was the view of patients and physician on co-location of a GPC and an emergency service.

**Results:**

404 patients and 488 physicians participated. 334 (82.7%) of all patients favoured a co-location. The major advantages were fast service (104, 25.7) and adequate referral (54, 13.4%). 237 (74%) of the GPs and 38 (95%) of the emergency physicians were in favour of a co-location. The major advantage was a more adequate referral of patients. 254 (79%) of the GPs and 23 (83%) of the emergency physicians believed that a co-location would lower the workload and waiting time and increase care quality (resp. 251 (78%), 224 (70%) and 37 (93%), 34 (85%).

**Conclusions:**

To close the expectation gap between GP’s, emergency physicians and to reach for high care quality, information campaigns and development of workflows are indispensable for a successful implementation of a co-location of primary and emergency care.

## Background

In Belgium, General Practitioner Cooperatives (GPC) aim to improve working conditions for out of hours primary care and to reduce the number of low acuity emergency visits by patients. In Belgium, about eighty GPCs have been established since 2003. These GPCs operate as walk-in centres for unplanned out of hours care and are staffed by the regional GPs. However, this investment has not led to a decrease in the number of emergency contacts. Moreover, the organization of the GPCs is not aligned to the emergency services: limited opening hours, no agreements with the emergency services on referral, located outside the hospital environment, insufficiently known by the population [1, 2].. There is also a low effective workload at a substantial number of GPCs, especially at night (e.g. the average number of contacts per night ranges from 1 to 4 consultations and from 1 to 3.5 home visits) [2].

Low acuity use of the emergency department (ED) is defined as use for non-urgent conditions or conditions that can be handled by a general practitioner (GP) [3, 4]. As compared to other countries, the number of low acuity visits is very high in Belgium [5]. Patients have free access to the ED where they expect to receive a fast, efficient and sophisticated service [6, 7]. Above, patients are more familiar with this type of out of hours or unplanned care than with the service offered by the GPCs [7, 8].

To address the problem of low acuity use of emergency services and to improve efficiency and productivity of the GPCs, the government installed the nationwide number 1733 for unplanned, non-live threatening out of hours care [9]. In this system, an operator directs the caller to the appropriate level of care: three levels of ambulance intervention, urgent or planned referral to out of hours primary care services or to planned care (https://www.health.belgium.be/en/health/need-call-physician -call-1733). These operators are trained to follow digital algorithms and protocols. The protocols are the result of a collaboration between academic partners, the Federal Public Service of Health and experts in general practice and in emergency medicine [9, 10].

Internationally, there is also a plea for a physical collaboration between the EDs and the GPCs for the organisation of unplanned care [11–13]. In this so-called co-location model, both services offer a single entrance to care with a joint triage to the appropriate care level following algorithms and protocols. Both services operate independently but maintain a high level of mutual consultation and referral.

This relatively new approach of a single entrance with joint triage to the appropriate care level requires support from all stakeholders and a bottom up development of operational procedures. In this research, we explored the view of patients, GPs and emergency physicians on the co-location of a GPC and an emergency service for unplanned care.

## Methods

The study was performed in a quantitative, cross section design. The primary research question was: what is the view of patients and physicians (GPs and emergency physicians) on the co-colocation of a GPC and an emergency service?

Both questionnaires were newly developed as the result of the findings of previous research, expert consult and the input of the advisory board of the federal taskforce [9, 14]. In a first round, after instruction and briefing by the taskforce to pre-define the themes, the team of researchers prepared the questions. The promotors of the research (BS, MS) and the general coordinator of the 1733-implementation project (CVDM) reviewed this draft version. In a second round, the revised questionnaires were presented to the task force and to the advisory board of the 1733-project. In a third round, the field-coordinator of the GPC checked the feasibility and acceptability of the third version. After this round, the questionnaires were finalized.

In the patient questionnaire, patients were asked to share their views on: accessibility of the national 1733 number, triage to the appropriate care level (emergency service, urgent or planned referral to out of hours primary care services or to planned care), the availability of speciality care and on the accessibility of the care services. The answer options were distributed on a 5-item Likert-scale (fully agree to fully disagree).

The physician questionnaire explored the view of the physicians on the concept and the organization of a co-location service for unplanned care: if they are in favour of the system and what are the expected advantages and disadvantages. Above, the participants rated seven statements addressing the following: ideas and expectations of co-location, time efficiency, care quality, practical conditions to collaborate and mastering of competences. This questionnaire used a 2-item Likert scale for the answer options (rather agree-rather not agree).

Recruitment took place in a representative region of Belgium. Indicators for representability were the characteristics of the ‘care zone’ (a federal framework to organize care) and demographic characteristics. Patients were recruited in three regional GPCs and in one emergency service. The local coordinators of the GPCs and of the emergency service were comprehensively briefed on the aim of the study and the recruitment of patients. These coordinators asked patients to consider participation, handed over the questionnaire in case of interest and provided support if required. The inclusion criteria for patients were domiciliation in the region, older than 18 and Dutch speaking or understanding. Physicians were recruited in advance through the local GP-organizations and hospitals. The inclusion criterion was ‘working as GP or as emergency physicians in an emergency department’.

Because patients were recruited on site, they were invited to complete a paper version of the questionnaire. The physicians received an electronic invitation of the questionnaire. The questionnaire was offered via the QualtricsXM-software on a secured server (KU Leuven). For univariate analyses we used Excel 2016 and for the multivariate analyses SAS 9.4. A multivariate logistic regression analyse was performed with ‘in favour of co-location’ as dependent variable for both datasets (patient and physician). The predicting variables were all background features of patients and physicians and their view on co-location. The predictors were added and omitted in a stepwise procedure. Odd ratios and 95% confident intervals were calculated for the independent, predicting variables.

The study took place between July 1 and December 312,019.

### Ethics approval and consent to participate

The study was approved by the Medical Ethical Board of the University Hospitals of Leuven under the number MP009864. Patient participants signed an informed consent after verbal briefing by the local coordinator. A more comprehensive information letter was available on simple request.

## Results

### Patients

404 patients completed the questionnaire. These patients visited on average 0.7 times a year a GP on duty and 0.5 times a year an emergency department. (Table 1).

**Table 1:** Characteristics of patients

	n(%)			n(%)		n(%)
**Age (years)**		**Visit < 6 months**			**Location GP on duty**	
18–30	90 (22,3)	GP			Own practice	7 (1,7)
31–45	125 (30,9)	0		13 (3,2)	GPC	317 (78,5)
46–65	136 (33,7)	1–4		199 (49,3)	- Independent	313 (77,5)
65+	53 (13,1)	≥5		189 (46,8)	- Next to ED	4 (1,0)
**Care region**		Blank		3 (0,7)	National number for unplanned care (1733°	5 (1,2)
Rural	117 (29,0)	**GP on duty**			Combination	4 (1,0)
GP	71 (17,6)	0		255 (63,1)	Own practice and GPC	2 (0,5)
GPC	46 (11,4)	1		84 (20,8)	1733 and GPC	2 (0,5)
Semi-urban	179 (44,3)	> 1		65 (16,1)	Don’t know	62 (15,3)
GP	149 (36,9)	**ED**			Blank	9 (2,2)
GPC	30 (19,8)	0		268 (66,3)		
Urban	108 (26,7)	1		102 (25,2)		
GP	80 (19,8)	> 1		34 (8,4)		
ED	28 (6,9)					

GP: general practitioner

GPC: General Practitioner Cooperatives

ED: emergency department

Overall, 334 (82.7%) of all patients favoured a co-location (Table 2). The expected advantages of this system were a fast service (104, 25.7%) and an adequate referral (54, 13.4%). The major advantages of the telephone triage were a fast service (108, 26.7%), reassurance (74, 18.3%), advice (52, 12.9%) and a reduction of unnecessary transfers (83, 20.5%) (Table 2). The major disadvantage of telephone triage was the risk on an inadequate operators’ assessment due to the lack of a clinical examination, a misinterpretation of symptoms and the lack of a patients’ file (resp. 30.4, 12.4 and 8.7%) (Table 2). When co-location implies that patients will always need a referral, then patients agreed with the system if the waiting time for unplanned care decreased (resp. 246, 60.9 and 259, 71.5 for GP and ED) (Table 3). Although for particular musculoskeletal problems or cutting wounds, patients (307, 76% and 306 75.7%) preferred a visit to the ED, most patients (353, 87.4%) had confidence in the competences of the GP (Table 2).

**Table 2:** Advantages/disadvantages of telephone advice and triage by GP and of co-location

	Telephone advice by GP					
**Advantage**		**n(%)**	**Disadvantage**			**n(%)**
Time to care			Wrong clinical assessment			
Fast service		108 (26,7)	No physical examination			123 (30,4)
24/7 available		8 (2,0)	No patient history			35 (8,7)
Telephone contact with physician			Limited anamnesis			50 (12,4)
Reassurance		74 (18,3)	Barriers in anamnesis due to language/understanding			4 (1,0)
Advice		52 (12,9)	Not specified			18 (4,5)
Assessment by physician		12 (3,0)				
Practical advantages			Not reassured			25 (6,2)
No extra transfer		83 (20,5)	Long waiting time			11 (2,7)
Cost saving		17 (4,2)	No prescriptions			10 (2,5)
Reduce workload for ED and GP		2 (0,5)	High responsibility			1 (0,2)
Other		7 (1,7)	Other			8 (2,0)
No advantage		13 (3,2)	No disadvantage			7 (1,7)
Blank		118 (29,2)	Blank			158 (39,1)
	**Co-location**					
**Options**	**n(%)**	**Advantages**	**n(%)**	**Disadvantages**		**n(%)**
Fully agree	90 (22,3)	Fast service	104 (25,7)	Further from home		19 (4,7)
Agree	244 (60,4)	No extra transfer	103 (25,5)	Parking/transport		3 (0,7)
Disagree	31 (7,7)	Correct referral	54 (13,4)	Longer waiting time Other		19 (4,7) 41 (10,2)
Fully disagree	6 (1,5)	Multi-disciplinary	29 (7,2)			
Blank	33 (8,2)	Well equipped setting	18 (4,5)			
**Telephone registration on co-location n (%)**						
**Options**	**Physician at telephone**	**Telephone advice**	**Operator assessment**		**GP assessment**	
Fully agree	100 (25,1)	159 (39,4)	47 (11,6)		101 (25,0)	
Agree	137 (34,3)	208 (51,5)	187 (46,3)		226 (55,9)	
Disagree	127 (31,8)	22 (5,4)	133 (32,9)		61 (15,1)	
Fully disagree	35 (8,8)	11 (2,7)	32 (7,9)		10 (2,5)	
Blank	5 (1,3)	4 (1,0)	5 (1,3)		6 (1,5)	
**Triage on co-location n (%)**						
**Options**	Correct referral operator	Ignore referral operator	No own initiative visit GP	No own initiative visit ED	No free choice	Adequate referral
Fully agree	83 (20,5)	16 (4,0)	57 (14,1)	86 (21,3)	55 (13,6)	167 (41,3)
Agree	266 (65,8)	90 (22,3)	189 (46,8)	203 (50,2)	210 (52,0)	164 (40,6)
Disagree	36 (8,9)	213 (52,7)	113 (28,0)	72 (17,8)	93 (23,0)	94 (23,3)
Fully disagree	13 (3,2)	71 (17,6)	33 (8,2)	24 (5,9)	26 (6,4)	20 (5,0)
Blank	6 (1,5)	14 (3,5)	12 (3,0)	19 (4,7)	20 (5,0)	167 (41,3)

**Table 3:** Characteristics of physicians

Years of experience	0- > 5y	171 (36)
	6- > 15y	114 (24)
	16- > 25y	70 (15)
	26- > 35y	75 (16)
	>35y	48 (10)
Gender	Gender neutral	1 (0)
	Male	236 (48)
	Female	249 (51)
Discipline	Other (management)	15 (3)
	ED resident	35 (7)
	GP	320 (66)
	GP resident	77 (16)
	GP	40 (8)
Type of practice GP	Other	12 (4)
	Duo	53 (17)
	Group	174 (54)
	Solo	71 (22)
	Local Medical Centre	9 (3)
Type of practice ED physician	Other	1 (3)
	Regional Hospital with co-location	9 (23)
	Regional hospital without co-location	s22 (55)
	University Hospital (without co-location)	8 (20)
Location GP on duty	Other	13 (3,3)
	Co-location	45 (11,3)
	Own practice	54 (13,7)
	GPC	285 (71,7)

Patients who favoured a co-location were patients who preferred a physician above an operator when calling 1733 (OR = 0,93 (95% CI 0,88 – 0,96). Supporters of co-location were also younger and were satisfied with a telephone advice (resp. OR = 0,98 (95% CI 0,98 – 0,99); OR = 1,25 (95% CI 1,14 – 1,38). Lastly, supporters of the co-location agreed with the impossibility of self-referral (resp. OR 1,25 (95% CI 1,15 – 1,37; OR = 1,11 (95% CI 1,01 – 1,22)).

### Physicians

488 physicians participated of which 320 (66%) GPs, 77 (16%) GP residents, 40 (8%) emergency physicians and 35 (7%) emergency residents. 232 (73%) of all GPs participated in an unplanned care GPC and 36 (11%) operated on co-location. (Table 3).

237 (74%) of the GPs and 38 (95%) of the emergency physicians were in favour of a co-location (Table 4). Supporters of the co-location (respectively 97/237 and 18/38), reported as major advantage a more adequate referral of patients. 254 (79%) of the GPs and 23 (83%) of the emergency physicians believed that a co-location would lower the workload. GPs and emergency physicians believed that waiting time would decrease and care quality would increase (resp. 251 (78%), 224 (70%) and 37 (93%), 34 (85%) (Table 4). GPs and emergency physicians believed that patient satisfaction would increase in the co-location system (resp. 217 (68%) and 30 (70%)) (Table 5). 104 (33%) GPs and 226 (65%) emergency physicians believed that GPs were sufficiently skilled to interpret the standard technical examinations (lab results, imaging) (Table 5). 212 (68%) GPs and 33 (83%) emergency physicians expected an increase in technical examinations prescribed by the GP in case of a co-location (Table 5). Finally, Physicians advocated an independent structure and organization of both services in a co-location system (resp. GP 238, 74%, ED physicians 25, 63%). Half of the ED-physicians (20, 50%) and 13 (4%) GPs preferred a 24/7 opening of the co-location. (Table 5).

**Table 4:** Major advantages when in favour of co-location by physician

Answer options	GP	ED physician	GP resident	ED resident
In favour of co-location	237 (84)	38 (95)	63 (82)	31 (89
Efficient care	44 (19)	10 (26)	13 (21)	3 (10)
Adequate referral	97 (41)	18 (47)	19 (30)	22 (71
Improvement of care quality	23 (10)	5 (13	6 (10)	3 (10)
Proximity of hospital for technical examinations	54 (23)	NA	23 (37)	2 (6)
Reduction of workload	7 (3)	2 (5)	NA	NA
More referrals to ED	5 (6)		1 (11)	
Low acuity protocols	7 (9)		2 (22)	
Insufficient confidence in system	7 (9)	0	NA	NA
Increase in workload	10 (13)		2 (22)	0
Decrease in independency	24 (30)	1 (50)	2 (22)	0
Overuse of technical examinations	11 (14)	NA	1 (11)	NA

Legend: Only fully completed records

**Table 5:** Perspectives of physicians on co-location

	Rather agree n (%)				Rather not agree n (%)			
Perspective	ED 40 (8)	GP 320 (66)	GPres 77 (16)	EDres 35 (7)	ED 40 (8)	GP 320 (66)	GPres 77 (16)	EDres 35 (7)
Decrease in workload	27 (68)	254 (79)	66 (86)	29 (83)	11 (28)	57 (18)	3 (4)	4 (11)
Improvement of care quality	34 (85)	224 (70)	56 (73)	30 (86)	4 (10	67 (21)	9 (12)	1 (3)
Decrease in time to treatment	37 (93)	251 (78)	62 (81)	31 (89)	1 (3)	59 (18)	7 (9)	2 (6)
Increase in patient satisfaction	30 (75)	217 (68)	54 (70)	27 (77)	7 (18)	80 (25)	12 (16)	5 (14) 217 (68)
Use of ED infrastructure by GP	20 (50)	200 (63)	57 (74)	20 (57)	18 (45)	100 (31	9 (12)	12 (34
GP competent technical ex.	26 (65)	NA	NA	23 (66)	12 (30)	NA	NA	8 (23)
GP interpretation technical ex.	NA	225 (70%)	48 (62)	NA	NA	72 (22)	16 (20)	NA
Meer technical ex.	5 (13)	79 (25)	12 (16)	4 (11)	33 (83)	219 (68)	53 (69)	28 (80)
Independency of services	25 (63)	238 (74)	57 (74)	18 (51)	13 (33)	60 (19)	13 (33)	13 (37)
Higher cost effectiveness	29 (73)	227 (71)	54 (70)	28 (80)	8 (20)	72 (23)	12 (16)	4 (11)
Multi-disciplinary consult	34 (85)	277 (87)	59 (77)	31 (89)	2 (5)	24 (8)	7 (9)	1 (3)
	**ED 40 (8)**		**GP 320 (66)**		**GPres 77 (16)**		**EDres 35 (7)**	
**Pre-triage**								
Other	1 (3)		10 (3)		2 (3)		3 (9)	
GP	4 (10)		43 (13)		6 (8)		4 (11)	
Trained personnel	8 (20)		148 (46)		27 (35)		9 (26)	
ED physician	6 (15)		6 (2)		0		1 (3)	
ED nurse	18 (45)		33 (10)		20 (26)		10 (29)	
	**ED 40 (8)**		**GP 320 (66)**		**GPres 77 (16)**		**EDres 35 (7)**	
**Opening hours**								
24/7	10 (29)		13 (4)		4 (5)		20 (50)	
Other	2 (6)		21 (7)					
Only out of hours	17 (49)		154 (48)		38 (49)		16 (40)	
From 6 pm till midnight	1 (3)		11 (3)		2 (3)		1 (3)	
Only Weekend	1 (3)		96 (30)		20 (26)		1 (3)	
**Committed to work in co-locatio**								
Part time	NA		15 (5)		8 (10)		NA	
Only duty hours	NA		215 (67)		49 (64)		NA	
Fulltime	NA		3 (1)		5 (6)		NA	
No	NA		65 (20)		2 (3)		NA	

Legend: Only fully completed records

Opponents of the system preferred to work full time in this service (OR 24,6, 95% CI 12,0-50,4). These opponents also believed that care quality would not improve (OR 0,030, 95% CI 0,015-0,057). They expected an increased number of technical examinations prescribed by the GPs (OR 2,6, 95% CI 1282-5156). Opponents predicted that the waiting time would not decrease in a co-location system (OR 0,292, 95% CI 0,164-0,522).

## Discussion

In this study, we explored the view of physicians and patients on the co-location of a GPC and an emergency service for unplanned care. The majority of patients was in favour of a co-location. More than two thirds of all patients believed that the benefit of co-location would be a decrease in waiting time. Although patients preferred to visit an ED for particular problems, the majority also had confidence in the competences of a GP. Co-location is particularly preferred by younger patients, patients who had confidence in a telephone operator and patients who were satisfied with a telephone advice.

The majority of ED-physicians and three quarter of the GPs were in favour of a co-location for unplanned care. Physicians believed that this system would benefit the adequacy of referral. They also expected a decrease in workload and waiting time and an improvement of care quality. Only one third of the GPs believed that they were sufficiently skilled to work in a co-location. ED physicians expected an increase in prescriptions of technical examinations by GPs. Only half of the ED physicians and a handful of GPs were in favour of a 24/7 opening of the co-location.

When patients use unplanned care services they are particularly concerned about waiting times, adequacy of referral and competences of the attending physician (and medical staff) [6, 8, 15]. The waiting time for unplanned care is a major concern in most health care systems [16]. Self-referring patients, understaffing and infrastructural limits are causing this problem [5, 8, 16]. Patients in our study were open for referral in case of unplanned care if performed by a trained telephone operator. From earlier research, we know that referrals made by the operator happen safely and adequately for all levels of care [9, 17–20]. When the adequacy of referring increases, the patient inflow for unplanned care, workload and waiting lists will decrease. In a co-location service, patients expect a reduction of transfers for further examination or follow up [7, 11, 12, 21–23]. Therefore, a particular point of attention in the organization of unplanned care is the accessibility of these services. Local government could play a role in supporting the accessibility of services but also in public campaigns to promote a correct use of unplanned care [10, 24]. Patients who expected a direct contact with a physician in case of an unplanned care need turned more often directly to their preferred level of care. In reality, this probably means that they will sign in to the ED [5, 6, 23]. This action will slow down the time to intervention for the index patient but also for correctly referred patients by interfering with the regular workflow. Finally, the older patients seemed less prepared to accept a referral to a co-location service since they probably preferred the familiarity with the current system [6, 25, 26].

Patients who visited the ED for particular problems also declared their confidence in the skills and competences of the GP. The link between these particular problems (wounds, musculoskeletal problems) and emergency care rather is the consequence of an image problem than of a rational believe that GPs are unable to deal with minor traumas [5, 22, 26–28]. Younger patients, patients who are less attached to self-referring or patients who trusted the advice of an operator were more likely to favour co-location. Indeed, patients who are more familiar with the current healthcare system are more likely to accept new systems [10, 29]. Repeated and comprehensible public campaigns play a major supporting role in the acceptance of a new system [10].

More ED physicians than GPs were in favour of a co-location. GPs fear that their input and expertise will be overruled in a hospital setting [8, 11, 30] Therefore, in a co-location care system, a strict flowchart and a role definition are very important [17, 23, 31]. Both groups confirmed that a co-location might improve the adequacy of referral, lower the workload and waiting times. These indicators have a high impact on health care outcomes and on patient satisfaction [6, 28]. To meet the GP’s concern of being overruled by hospital specialists, the infrastructure and working conditions could be customized to the needs of primary care. An on the spot training of GPs in prescribing and interpreting technical examinations might also reduce the number of unnecessary or inadequate technical examinations [11]. Both groups claimed a certain level of independency. A robust corporation agreement could align the structural independency with the collaboration in patient care [7, 29]. The finding that GPs and ED physicians disagreed on the opening hours of the co-location might be a first discussion point. Other than the ED-physicians, GPs operate independently in their own practice during regular working hours. Day shifts in a 24/7 service imply a reorganization of their practice schedule. ED-physicians already work in a 24/7 schedule which implies that the 24/7 system will not affect their working conditions. Physicians who rejected the concept of a co-location preferred at the same time a 24/7 system. Indeed, in a 24/7 service, duty scheduling probably will be performed under better conditions.

The major strength of this study lies in the population reached. A representative part of the target population participated during the test period. This is also the first study giving insights in the bottom up construction of collaboration between ED and primary care for unplanned care. The questionnaires were constructed with the aid of the task force of unplanned care in Belgium. This group of experts built the foundations for the reorganisation of unplanned care.

A participation bias cannot be ruled out since most questionnaires were completed by patients visiting the GP. The population frequently visiting the ED might be absent in this study. Others, most patients in Belgium have a GP and visit the GP at least once a year. Second, the general patient characteristics were representative. Nevertheless, it would be interesting to recruit a proportional number of patients visiting the ED and compare them to the group of GP-visitors.

Second, no distinction was made between patients who completed the questionnaire for themselves or third parties accompanying a patient. However, in case of a third party, this person will also decide on the level of care needed. However, indeed, we cannot rule out that more vulnerable or frail patient groups have other ideas about unplanned care and therefor need another, more specific approach.

Finally, this study did not focus on objective outcomes as cost effectiveness, care quality and patient and physician satisfaction. The study only relied on the assumptions, expectations and insights of the participants. This approach might also be a strength since it uncovers the barriers that need to be addressed in further research or during implementation. To underpin the statements and assumptions made by patients and physicians, further research should certainly focus on a cost-benefit analysis.

## Conclusions

Patients and physicians favour a co-colocation of ED and GPC for unplanned care under certain conditions and circumstances. In particular, both parties expected a decrease in waiting time. Patients still express their preferences to visit an ED but the majority also seemed to have confidence in a GP. Co-location might meet these concerns and combine and rationalize the offer and use of services. Therefore, further research should focus on cost effectiveness, care quality and patient and physician satisfaction in a concrete co-location site.

## Data Availability

The datasets generated and analysed during the current study are available in the Dataset Co-location repository, https://kuleuven-my.sharepoint.com/:x:/g/personal/birgitte_schoenmakers_kuleuven_be/EcKEZ_9-mwFPoRWcDAIG_kgB1b7tTp52epkm1EMGNLxxhw?e=jTpVk7

## Abbreviations

GPC: General Practitioner Cooperatives
ED: Emergency department
GP: General practitioner

## References

[1] Philips H, Michiels B, Coenen S, Remmen R (2014). Reducing inappropriate a&E attendances. Br J Gen Pract.

[2] Van den Heede KDC, Devriese S, Baier N, Camaly O, Depuijdt E, Geissler A, Ghesquiere A, Misplon S, Quentin W, Van Loon C, Van de Voorde C (2016). Organizatie en financiering van spoeddiensten in België: huidige situatie en opties voor hervorming Health Services Research (HSR) Brussel: Federaal Kenniscentrum voor de Gezondheidszorg (KCE) 2016 KCE Reports 263As D/2016/10273/21.

[3] Uscher-Pines L, Pines J, Kellermann A, Gillen E, Mehrotra A (2013). Emergency department visits for nonurgent conditions: systematic literature review. Am J Manag Care.

[4] Young GP, Wagner MB, Kellermann AL, Ellis J, Bouley D (1996). Ambulatory visits to hospital emergency departments. Patterns and reasons for use. 24 hours in the ED study group. JAMA..

[5] Brasseur E, Gilbert A, Servotte JC, Donneau AF, D'Orio V, Ghuysen A. Emergency department crowding: why do patients walk-in? Acta Clin Belg. 2019:1–7. 10.1080/17843286.2019.1710040. 10.1080/17843286.2019.1710040 31886742

[6] Ghazali DA, Richard A, Chaudet A, Choquet C, Guericolas M, Casalino E. Profile and Motivation of Patients Consulting in Emergency Departments While not Requiring Such a Level of Care. Int J Environ Res Public Health. 2019;16(22). 10.3390/ijerph16224431 PMC6888183 31726697

[7] Colliers A, Remmen R, Streffer ML, Michiels B, Bartholomeeusen S, Monsieurs KG, Goris J, Coenen S, Verhoeven V, Philips H (2017). Implementation of a general practitioner cooperative adjacent to the emergency department of a hospital increases the caseload for the GPC but not for the emergency department. Acta Clin Belg.

[8] Rutten M, Vrielink F, Smits M, Giesen P (2017). Patient and care characteristics of self-referrals treated by the general practitioner cooperative at emergency-care-access-points in the Netherlands. BMC Fam Pract.

[9] Van der Mullen CQH, Van Baelen S, Crits T, Wuyts J, Sabbe M (2017). De patiënt met een niet-planbare zorgvraag naar het gepaste zorgniveau verwijzen: nieuwe 112-1733 geïntegreerde telefonische triage- en regulatieprotocollen. Tijdschrift voor Geneeskunde.

[10] Philips H, Verhoeven V, Morreel S, Colliers A, Remmen R, Coenen S, van Royen P (2019). Information campaigns and trained triagists may support patients in making an appropriate choice between GP and emergency department. Eur J Gen Pract.

[11] van Veelen MJ, van den Brand CL, Reijnen R, van der Linden MC (2016). Effects of a general practitioner cooperative co-located with an emergency department on patient throughput. World J Emerg Med.

[12] van Gils-van Rooij ES, Yzermans CJ, Broekman SM, Meijboom BR, Welling GP, de Bakker DH (2015). Out-of-hours care collaboration between general practitioners and hospital emergency departments in the Netherlands. J Am Board Fam Med.

[13] Kool RB, Homberg DJ, Kamphuis HC (2008). Towards integration of general practitioner posts and accident and emergency departments: a case study of two integrated emergency posts in the Netherlands. BMC Health Serv Res.

[14] Philips H, Remmen R, Van Royen P, Teblick M, Geudens L, Bronckaers M (2010). What's the effect of the implementation of general practitioner cooperatives on caseload?. Prospective intervention study on primary and secondary care BMC Health Serv Res.

[15] Ebert JF, Huibers L, Lippert FK, Christensen B, Christensen MB (2017). Development and evaluation of an "emergency access button" in Danish out-of-hours primary care: a study protocol of a randomized controlled trial. BMC Health Serv Res.

[16] Poole R, Gamper A, Porter A, Egbunike J, Edwards A (2011). Exploring patients' self-reported experiences of out-of-hours primary care and their suggestions for improvement: a qualitative study. Fam Pract.

[17] Brasseur E, Servotte JC, Donneau AF, Stipulante S, d'Orio V, Ghuysen A (2019). Triage for out-of-hours primary care calls: a reliability study of a new French-language algorithm, the SALOMON rule. Scand J Prim Health Care.

[18] Plat FM, Peters YAS, Loots FJ, de Groot CJA, Eckhardt T, Keizer E, Giesen P (2018). Ambulance dispatch versus general practitioner home visit for highly urgent out-of-hours primary care. Fam Pract.

[19] Smits M, Keizer E, Ram P, Giesen P (2017). Development and testing of the KERNset: an instrument to assess the quality of telephone triage in out-of-hours primary care services. BMC Health Serv Res.

[20] Wheeler SQ, Greenberg ME, Mahlmeister L, Wolfe N (2015). Safety of clinical and non-clinical decision makers in telephone triage: a narrative review. J Telemed Telecare.

[21] Van den Heede K, Van de Voorde C (2016). Interventions to reduce emergency department utilisation: a review of reviews. Health Policy.

[22] Chalder M, Montgomery A, Hollinghurst S, Cooke M, Munro J, Lattimer V, Sharp D, Salisbury C (2007). Comparing care at walk-in centres and at accident and emergency departments: an exploration of patient choice, preference and satisfaction. Emerg Med J.

[23] Huibers L, Thijssen W, Koetsenruijter J, Giesen P, Grol R, Wensing M (2013). GP cooperative and emergency department: an exploration of patient flows. J Eval Clin Pract.

[24] Morreel S, Philips H, Verhoeven V. Self-triage at an urgent care collaboration with and without information campaign. Journal of emergency management (Weston, Mass). 2019;17(6):511–6. 10.5055/jem.2019.0443 31903540

[25] Philips H, Remmen R, De Paepe P, Buylaert W, Van Royen P (2010). Out of hours care: a profile analysis of patients attending the emergency department and the general practitioner on call. BMC Fam Pract.

[26] Chmiel C, Huber CA, Rosemann T, Zoller M, Eichler K, Sidler P, Senn O (2011). Walk-ins seeking treatment at an emergency department or general practitioner out-of-hours service: a cross-sectional comparison. BMC Health Serv Res.

[27] O'Kelly FD, Teljeur C, Carter I, Plunkett PK (2010). Impact of a GP cooperative on lower acuity emergency department attendances. Emerg Med J.

[28] Boeke AJ, van Randwijck-Jacobze ME, de Lange-Klerk EM, Grol SM, Kramer MH, van der Horst HE (2010). Effectiveness of GPs in accident and emergency departments. Br J Gen Pract.

[29] Smits M, Rutten M, Keizer E, Wensing M, Westert G, Giesen P (2017). The development and performance of after-hours primary Care in the Netherlands: a narrative review. Ann Intern Med.

[30] Cowling TE, Ramzan F, Ladbrooke T, Millington H, Majeed A, Gnani S (2016). Referral outcomes of attendances at general practitioner led urgent care centres in London, England: retrospective analysis of hospital administrative data. Emerg Med J.

[31] Smits M, Hanssen S, Huibers L, Giesen P (2016). Telephone triage in general practices: a written case scenario study in the Netherlands. Scand J Prim Health Care.

